# NanoConfinementChat: A Domain-Specific Large Language Model for Mechanistic Interpretation of Nanoconfined Water and Ion Transport in Membrane Nanochannels

**DOI:** 10.3390/membranes16100319

**Published:** 2026-09-26

**Authors:** Shuhan Liu, Yao He, Youyang Liu, Ziyi Yan, Xinyu Zhang, Yuxin Xiao, Zhen Li, Ning Wei

**Affiliations:** Jiangsu Key Laboratory of Advanced Food Manufacturing Equipment and Technology, Jiangsu Province Engineering Research Center of Micro-Nano Additive and Subtractive Manufacturing, Institute of Advanced Technology, School of Mechanical Engineering, Jiangnan University, Wuxi 214122, China

**Keywords:** membrane nanochannels, nanoconfined transport, water and ion transport, ion selectivity, large language models, retrieval-augmented generation, membrane transport

## Abstract

Nanoconfined water and ion transport determines permeability and selectivity in membrane nanochannels, yet the relevant mechanisms are dispersed across material systems, molecular simulations, and theoretical studies, hindering systematic mechanism-oriented knowledge synthesis. Here, we develop NanoConfinementChat, a domain-specific large language model (LLM) for analyzing transport mechanisms in ion-selective membrane nanochannels. A curated corpus of 518 papers was used to construct 8500 mechanism-oriented instruction–answer pairs for supervised fine-tuning (SFT) of Llama-3.1-8B-Instruct using low-rank adaptation (LoRA). A retrieval-augmented generation (RAG) module was further integrated for literature grounding and source tracing, and model performance was assessed using a 90-question gold-point benchmark. Without RAG, NanoConfinementChat achieved an average benchmark score of 2.144 and a major scientific error rate of 2.22%, compared with 11.11–13.33% for the two baseline models. Case studies showed improved mechanistic interpretation of ordered water transport, dehydration-driven ion exclusion, graphene oxide interlayer-spacing effects, electrostatic exclusion, and evidence requirements for interfacial slip. Domain fine-tuning provided the primary performance gain, while RAG improved source traceability. The framework offers a lightweight approach for evidence-grounded interpretation of structure–mechanism–transport relationships in membrane nanochannels.

## 1. Introduction

Ion-selective membrane nanochannels represent a foundational paradigm across advanced fluidics, molecular separations, and energy conversion systems [1,2,3,4,5,6,7,8,9]. The macroscopically observed mass-transfer performance in these architectures is fundamentally governed by the selective transport of molecules and ions across subnanometer or angstrom-scale spatial confinements. Platforms such as carbon nanotubes (CNTs), graphene and graphene oxide (GO) laminates, two-dimensional slit channels, and biomimetic pores have emerged as excellent model systems for investigating and regulating transport flux and multi-species selectivity [6,10,11,12,13,14,15,16,17,18,19,20,21,22].

In these highly confined environments, nanoscale mass transfer is no longer adequately described by classical continuum mechanics or basic geometric sieving. Instead, transport kinetics and separation selectivities are cooperatively governed by a non-continuum interplay of hydration-shell distortion, ion dehydration energy penalties, steric confinement, electrostatic exclusion, interfacial slip, low-friction single-file fluid configurations, wall–fluid interactions, and pore-entry free-energy barriers. Consequently, identical macroscopic mass-transfer outcomes—such as anomalous flux enhancement or strict target exclusion—may stem from completely distinct microscopic mechanisms depending on channel geometry, surface charge density, interfacial chemistry, ion species, and specific boundary or driving conditions [23,24,25,26,27,28,29,30,31,32,33,34,35,36,37].

This mechanism-intensive and condition-dependent nature renders knowledge organization and synthesis in nanofluidic mass transfer exceptionally challenging. Critical mechanistic insights remain heavily fragmented across interfacial chemistry, condensed matter physics, fluid dynamics, and atomistic molecular dynamics (MD) simulations [6,14,32]. Such disciplinary dispersion frequently leads to terminological ambiguities and conceptual conflations during transport interpretation. For instance, steric exclusion and thermodynamic dehydration barriers both dictate mass selectivity but arise from fundamentally different physical constraints [23,26]; similarly, interfacial slip length and low-friction configurations are intimately linked to transport propagation but do not reflect identical microscopic processes [32,33,34,35].

Large language models (LLMs) are increasingly being leveraged to bridge such interdisciplinary knowledge gaps by transforming from general-purpose text processors into domain-specific scientific assistants [38,39,40,41]. However, general-purpose LLMs exhibit systemic failure modes when task-oriented reasoning over confined mass-transfer phenomena is required. They are prone to oversimplifying transport mechanics to simple geometric sieving while ignoring thermodynamic hydration penalties; explaining flux dynamics purely via surface hydrophilicity while missing interfacial slip lengths or single-file motion characteristics; and completely overlooking whether reported MD simulation conclusions are strictly bounded by force-field selection, driving forces, thermostats, or specific boundary parameters [41,42]. These intrinsic limitations impede their utility as reliable assistants for mechanism-aware literature mining and scientific reasoning in nanofluidics and transport science.

To address these challenges, we develop a domain-specific large language model tailored for mechanism-aware reasoning of nanoscale mass-transfer phenomena in ion-selective membrane nanochannels. This framework is specifically engineered to move beyond surface-level text indexing by reconstructing compact structure–mechanism–transport reasoning chains and auditing hidden evidence gaps within literature statements [43]. Concretely, this work delivers four principal contributions: (1) We constructed a curated nanoconfined mass-transfer corpus covering ion transport, interfacial friction, dehydration barriers, and pore-entry effects; (2) we generated 8500 mechanism-oriented instruction–answer pairs to encode structure–mechanism–transport reasoning; (3) we developed a domain-specific large language model by LoRA-based SFT and RAG to support evidence-grounded analysis of transfer mechanisms; (4) we evaluated the model using a gold-point benchmark focused on mechanism correctness, condition dependence, and scientific error reduction.

## 2. Materials and Methods

### 2.1. Study Design and Domain Adaptation

In this study, we developed a domain-specific large language model, hereafter named NanoConfinementChat, for the mechanism-aware reasoning of nanoscale mass transfer in ion-selective membrane nanochannels. The study design follows the domain-specific scientific LLM workflow exemplified by GrapheneChat, in which curated domain corpora, supervised fine-tuning, retrieval-augmented generation, and dedicated benchmarking are combined to construct specialized research assistants. In this work, this workflow was adapted to a distinct scientific setting: nanoconfined transport in ion-selective nanochannels. The methodological novelty lies in constructing a mechanism-driven framework tailored for nanoscale mass transfer, which includes corpus construction, mechanism-oriented instruction design, retrieval grounding, gold-point evaluation, and transport-relevant case validation, rather than in proposing a new LLM architecture. This design was intended to examine whether a lightweight open-source model could be adapted to a mechanism-intensive membrane field where mass flux, ion selectivity, permeation barriers, and structure–transport relationships are central research questions. The overall adaptation strategy is summarized in Figure 1 and Table 1.

The overall workflow consisted of six steps: literature retrieval and screening, domain taxonomy construction, instruction–answer generation and filtering, LoRA-based supervised fine-tuning, retrieval-augmented literature grounding, and benchmark-based evaluation. These steps were organized to address the central challenge of mechanism-resolved interpretation rather than information retrieval alone. Specifically, the workflow was adjusted to emphasize mass-transfer nanoconfinement, including water/ion transport mechanisms, permeability–selectivity relationships, simulation-supported evidence, and condition-dependent structure–transport reasoning. The target capabilities included mechanism explanation, structure–property reasoning, method/simulation interpretation, and parameter judgment; at the evaluation level, concept distinction, condition-dependent reasoning, literature-oriented analysis, and comparison tasks were further assessed.

### 2.2. Literature Retrieval, Screening, and Domain Taxonomy

The literature corpus served as a common knowledge source for supervised fine-tuning and retrieval-augmented generation. The literature search was conducted using the OpenAlex API (application programming interface) on 11 April 2026. The search covered papers published between 2015 and 2025, and retrieved records were required to have a DOI (digital object identifier). The 2015 to 2025 publication window defines a bounded recent-literature corpus. The upper limit corresponds to the last complete calendar year before the April 2026 search. The lower limit is a practical scope restriction and does not imply that foundational pre-2015 studies are unimportant. Earlier papers cited for scientific background are distinct from the retrieved corpus. This restriction may underrepresent foundational evidence and its original conditions. Tables S1 and S2 describe the search scope and topical taxonomy, but a quantitative paper-level temporal and topical distribution is not reported. Expanding historical coverage and documenting these distributions remain necessary future work. Search results were ranked by citation count to prioritize influential studies. The query strategy combined four groups of terms covering nanoconfined-transport concepts, water/ion transport descriptors, mechanism- and simulation-related terms, and representative material systems. Representative terms included “nanoconfined transport”, “single-file water”, “Å-scale channel”, “water transport”, “ion transport”, “molecular dynamics”, “free energy”, “energy barrier”, “slip length”, “carbon nanotube”, “graphene oxide”, “aquaporin”, and “nanochannel”. Irrelevant topics such as photocatalysis, batteries, supercapacitors, sensors, electrodes, solar cells, therapy, cancer, imaging, robotics, actuators, and alloys were explicitly excluded. The full query list is provided in the Supporting Information.

The retained corpus was further organized into a mechanism-oriented nanoconfined-transport knowledge space, drawing on representative studies of CNT transport, graphene and GO membranes, biomimetic channels, nanofiltration selectivity, dehydration barriers, and nanofluidic friction [6,10,11,12,13,14,15,16,17,18,19,20,21,22,23,24,25,26,27,28,29,30,31,32,33,34,35,36,37], as summarized in Figure 2. This knowledge space connects membrane nanochannel systems, transport mechanisms, and evidence or analysis methods, providing the basis for subsequent instruction generation, retrieval grounding, and benchmark design.

Approximately 1000 candidate papers were collected before screening. The screening was performed in two stages. First, papers were classified according to their relevance to nanoconfined water/ion transport as highly relevant, moderately relevant, weakly relevant, or irrelevant. General membrane-separation papers without nanoscale mechanistic discussion, application-oriented engineering papers without physical interpretation, broad materials papers unrelated to water/ion transport under confinement, and papers from excluded fields were removed. Second, the highly and moderately relevant papers were further evaluated according to scientific quality, representativeness, mechanistic depth, and relevance to theoretical analysis, molecular simulation, free-energy analysis, or structure–property interpretation. After this two-stage screening, 518 papers were retained as the final domain corpus.

To organize the corpus, a three-level domain taxonomy was defined, including material-system labels, mechanism labels, and method/evidence labels. Material-system labels covered carbon nanotube channels, graphene and graphene oxide membranes, two-dimensional layered membranes, interlayer nanochannels, nanopores, subnanometer or Å-scale channels, aquaporins, and biomimetic water channels. Mechanism labels included dehydration barriers, hydration-shell distortion, steric exclusion, interfacial slip, low interfacial friction, wall–fluid interactions, electrostatic exclusion, hydrogen-bond network rearrangement, single-file water transport, free-energy or potential of mean force (PMF) barriers, structure–property relationships, water–ion transport decoupling, and entry or partitioning barriers. Method/evidence labels included molecular dynamics simulation, atomistic simulation, density functional theory (DFT) or first-principles calculation, theoretical modeling, free-energy calculation, PMF analysis, diffusion coefficient analysis, water flux/permeability analysis, ion rejection analysis, force-field-dependent interpretation, and literature-based mechanism interpretation. The complete taxonomy is listed in the Supporting Information.

### 2.3. Instruction–Answer Generation and Dataset Filtering

All selected papers were converted into plain-text documents before instruction–answer generation. The main text was extracted from each PDF, while references, repeated headers and footers, redundant formatting characters, and other non-informative fragments were removed. The extracted texts were normalized and length-controlled to improve compatibility with batch inference while retaining mechanistic descriptions, simulation conditions, transport parameters, and structure–property discussions. The full data construction and filtering workflow is summarized in Figure 3.

Instruction–answer pairs were generated using the Qwen Batch API with qwen3.5-plus. A domain-specific prompt template was designed to generate mechanism-oriented, structure–property, method/simulation, concept-distinction, and literature-oriented QA (question and answer) pairs from the cleaned full texts. The prompt required the answers to remain grounded in the source text and to prioritize physical mechanisms, simulation interpretation, key transport parameters, and structure–property relationships, rather than generic paper summaries. The prompt template is provided in the Supporting Information.

The raw QA outputs were not directly used for training. Valid QA pairs were first parsed and converted into the standard LLaMA-Factory format with instruction, input, and output fields. Empty questions or answers, extremely short answers, duplicated questions, and malformed entries were removed. A second filtering step was then applied to examine logic consistency between each question and its answer. A sample was retained only when the answer directly addressed the question and when the object, mechanism, parameter, condition, or conclusion was consistent between the question and the answer. Samples were removed when the answer was off-topic, only superficially overlapped with the question, lacked essential information, contained semantic mismatch, or was overly generic.

In the V2.5 filtering stage, 9561 candidate QA pairs were processed, and 8500 instruction–answer pairs were retained as the final supervised fine-tuning dataset. Thus, 1061 candidate samples were removed or not included in the final training set. Based on directly verifiable top-level labels in the final SFT file, mechanism explanation accounted for 2750 samples (32.35%), method/simulation interpretation for 1900 samples (22.35%), structure–property relationship for 1550 samples (18.24%), and parameter judgment for 1450 samples (17.06%). Generic samples accounted for 800 samples (9.41%), and samples labeled as “not mentioned” accounted for 50 samples (0.59%). Together, the four domain-oriented categories accounted for 90.00% of the final dataset. Concept distinction and literature-oriented reasoning were encouraged during prompt design and later evaluated in the benchmark, but they were not annotated as independent top-level labels in the final SFT file.

### 2.4. LoRA-Based Supervised Fine-Tuning

Llama-3.1-8B-Instruct was selected as a practical backbone for locally deployable domain adaptation using the reported single-GPU LoRA workflow. The qwen3.5-plus model was used through the Qwen Batch API for instruction-data generation, a separate role from the fine-tuning backbone. The present study did not perform a systematic backbone-selection comparison. Although Qwen2.5-7B-Instruct achieved a higher score before domain-specific SFT, its performance after comparable fine-tuning remains untested. The results do not establish that Llama-3.1-8B-Instruct is the optimal backbone, and comparisons with other similarly sized models remain future work. Fine-tuning was performed using LLaMA-Factory with low-rank adaptation. The objective was to align the base model with the terminology, mechanisms, and reasoning patterns of nanoconfined transport rather than to improve general open-domain conversational ability.

The supervised fine-tuning objective was the standard token-level autoregressive cross-entropy loss:

(1) $$L_{\mathrm{SFT}}=-\sum_{t=1}^{T}\log p_{\theta }(y_{t}|x,y_{<t})$$

where x denotes the input instruction, $y_{t}$ is the target token at decoding step t, $y_{<t}$ represents the preceding target tokens, and $p_{\theta }$ is the conditional probability distribution of the fine-tuned model.

The main hyper-parameters adopted during fine-tuning are summarized in Table 2. The optimized instruction dataset was registered in the LLaMA-Factory data configuration and used for LoRA-based SFT. Training used the llama3 template, a maximum sequence length of 2048 tokens, and LoRA applied to all target modules. The learning rate was set to 5 × 10^−5^, with a cosine scheduler and a warmup ratio of 0.1. The model was trained for 3 epochs with a per-device batch size of 2 and gradient accumulation steps of 4, resulting in an effective batch size of 8. FP16 (16-bit floating point) mixed precision, paged AdamW 8-bit optimization, and gradient checkpointing were used to reduce memory consumption. LoRA rank, alpha, and dropout followed the LLaMA-Factory default settings, namely 8, 16, and 0.0, respectively.

LoRA fine-tuning updated 20,971,520 trainable parameters out of 8,051,232,768 total parameters, corresponding to 0.2605% of the model parameters. Training was conducted on a single NVIDIA A10 GPU (graphics processing unit) and took approximately 1 h, 11 min and 54 s. The final training loss after 3 epochs was 1.4692. After training, the LoRA adapter was merged or deployed with the base model as NanoConfinementChat for downstream inference, RAG based question-answering, and benchmark evaluation.

### 2.5. RAG-Based Literature Grounding and Source Tracing

Fine-tuning enables the model to internalize domain-specific response patterns, but it does not solve the problem of source traceability. Therefore, a retrieval-augmented generation module was integrated to provide literature-grounded responses. This step is important for nanoconfined transport because mechanistic conclusions often depend on specific conditions, such as pore size, interlayer spacing, ion species, solution concentration, surface charge, force-field selection, driving force, and boundary conditions.

The RAG corpus was built from the curated PDF collection. Each paper was parsed page by page and segmented into overlapping text chunks with a chunk size of 800 characters and an overlap of 150 characters. Each chunk retained metadata including paper ID, article title, PDF filename, source path, and page number, enabling chunk-level and page-level source tracing. The text chunks were embedded using DashScope text-embedding-v4 and indexed in a persistent ChromaDB vector database.

During retrieval, the user query was embedded into the same vector space and compared with stored literature chunks using cosine similarity:

(2) $$s(q,d_{i})=\frac{e_{q}\cdot e_{d_{i}}}{\left\|e_{q}\|\|e_{d_{i}}\right\|}$$

where $e_{q}$ denotes the embedding vector of the query and $e_{d_{i}}$ denotes the embedding vector of the i-th literature chunk.

The offline testing pipeline used top-k = 4 retrieval, while a lightweight web interface used top-k = 1 for faster source-grounded response generation. Retrieved chunks were inserted into a source-grounded prompt together with the user query. The prompt instructed the model to use only information supported by the retrieved snippets, state uncertainty when evidence was incomplete, avoid fabricating papers, page numbers, mechanisms, or conclusions, and provide citations in the format [Source: Article Title, Page X]. The complete RAG implementation details and generation parameters are provided in the Supporting Information. The benchmark comparison in Section 3.2 evaluated the parameterized SFT model without RAG to isolate the effect of domain fine-tuning. The RAG module was evaluated separately as a source-traceable literature-grounding function in Section 3.4.

### 2.6. Benchmark Evaluation and Ablation Settings

A customized benchmark was constructed to evaluate whether NanoConfinementChat acquired the intended domain capabilities for mechanism-aware nanoconfined transport analysis. General-purpose benchmarks were not used as the main evaluation tool because they do not directly test the mechanism-intensive reasoning required in desalination-related nanoconfined transport research. The benchmark contained 90 English questions, each with a unique ID, module label, subtype, question text, and predefined gold points. The task modules included mechanism explanation, concept distinction, condition reasoning, literature/simulation interpretation, structure–property relationship, comparison, method-oriented reasoning, and parameter judgment. These categories were selected to examine whether a model could recover core physical mechanisms, distinguish related concepts, connect structural changes to transport responses, identify missing mechanistic evidence in literature-style statements, and select appropriate transport parameters.

The benchmark was maintained as an independent evaluation file and was not included in the SFT training dataset. However, the training data and benchmark were constructed within the same curated literature collection, and source-paper independence was not enforced. Duplicate removal within the SFT dataset does not establish the absence of training-test overlap. A systematic analysis of exact or semantic overlap across the two datasets has not been performed. The no-RAG evaluation therefore assesses performance on the present in-domain benchmark and does not establish generalization independent of corpus memorization. NanoConfinementChat, the original Llama-3.1-8B-Instruct base model, and Qwen2.5-7B-Instruct were evaluated on the same benchmark using the same prompt structure, decoding settings, maximum output length, and output format. The benchmark comparison evaluated the parameterized models without RAG to isolate the effect of domain fine-tuning. The RAG module was evaluated separately as a source-traceable literature-grounding function in Section 3.4. During dataset construction, duplicated questions and malformed entries were removed from the SFT data. The benchmark was designed to test mechanism explanation, concept distinction, condition reasoning, literature/simulation interpretation, and structure–property analysis rather than memorization of training examples.

Each benchmark question has three predefined gold points, which serve as reference elements for scoring the expected scientific content. The complete question texts and gold points are provided in newest test_90.jsonl (Table S8, Section S8b). The scoring rules and required output fields are specified in Table S7. The deepseek-chat judge assigns one point for each covered gold point, accepts scientifically equivalent wording and clearly implied coverage, and applies a single one-point penalty when the binary major-error indicator equals 1. The major-error criterion covers clear scientific error, conceptual confusion, mechanism reversal, severe off-topic reasoning, and misleading core judgment. These are criteria for assigning the binary indicator; separate frequencies for each error category were not assessed.

For each response, the raw score was calculated as:

(3) $$S_{i}^{\mathrm{raw}}=\sum_{k=1}^{K_{i}}I(g_{ik})-\lambda I(e_{i})$$

where $S_{i}^{raw}$ is the raw score for question i, $K_{i}$ is the number of predefined gold points, $I(g_{ik})$ indicates whether the k-th gold point was correctly covered, $I(e_{i})$ indicates whether a major scientific error was present, and λ is the penalty weight for a major scientific error. In this study, λ was set to 1. The final score $S_{i}$ was clipped at zero:

(4) $$S_{i}=\max(S_{i}^{raw},0)$$

The final score is clipped at zero and ranges from 0 to 3.

Condition dependence is represented in the question wording and its reference gold points. For example, NCT_056 asks whether stronger water ordering always implies faster transport; its gold points require rejection of this universal claim and recognition that ordering may support concerted transport but can also increase trapping, friction, or entropy loss. This reference illustrates the intended treatment of conditional mechanisms. The reported evaluation does not include a separate quantitative audit of condition consistency or establish that the automatic judge handles all condition-dependent claims correctly.

For the benchmark results, 95% confidence intervals (CIs) of the overall average score, module-level average score, and major scientific error rate were estimated by non-parametric bootstrap resampling over benchmark questions with 10,000 iterations and a fixed random seed of 42. To provide an additional check on the automatic scoring protocol, a stratified random subset of 11 benchmark questions was selected for expert manual review. For each selected question, the responses from the three evaluated models were anonymized and reviewed using the same gold-point coverage and major scientific error criteria. The documented validation consisted of automatic scoring of the full benchmark and a supplementary review of anonymized responses to 11 stratified questions by a domain expert using the same scoring criteria (Table S12). This assessment does not constitute broad external validation. Independent ratings from multiple human annotators are not available in the reported record, so inter-annotator agreement cannot be estimated. The Supplementary Materials provide the reference points and scoring outputs, but do not document item-level reference authorship or a multi-expert adjudication process for establishing the gold points.

To further examine the contribution of different components, ablation settings were designed to compare the base model, the base model with RAG, the SFT model without RAG, and the SFT model with RAG. These settings were used to distinguish the effects of domain fine-tuning and retrieval grounding on benchmark performance.

## 3. Results and Discussion

### 3.1. Mechanism-Oriented Dataset and Evaluation Design

The transport-specific nature of NanoConfinementChat is reflected in the coordinated design of its curated corpus, supervised fine-tuning dataset, and benchmark. As summarized in Figure 2, Figure 3 and Figure 4, these components were not intended to form a generic membrane-science question-answering resource. Instead, they were organized around the central challenge of this study: mechanism-resolved interpretation of nanoconfined water and ion transport for ion-selective nanochannels.

As shown in Figure 2, the curated corpus was structured around representative nanoconfined membrane systems, including carbon nanotube channels, graphene nanopores, two-dimensional layered membranes, biomimetic channels, and subnanometer or Å-scale confinements. These systems are directly related to the regulation of water permeability, ion selectivity, ion exclusion, and permeability–selectivity trade-off. More importantly, the corpus was structured around mechanism and evidence dimensions, including dehydration barriers, hydration-shell distortion, steric confinement, electrostatic exclusion, interfacial slip, wall–fluid interactions, PMF barriers, diffusion-related quantities, and simulation-based evidence. This organization provided the knowledge basis for training and evaluating a model that must connect material structure, transport mechanism, and supporting evidence.

The SFT dataset further reflects this mechanism-oriented design. As illustrated in Figure 3, the final 8500 samples were obtained after multi-stage cleaning and logic-consistency screening. Mechanism explanation, method/simulation interpretation, structure–property relationship, and parameter judgment collectively accounted for 90.00% of the final dataset. This distribution indicates that fine-tuning primarily exposed the model to causal transport reasoning, simulation-supported interpretation, and parameter-aware judgment, rather than generic scientific summarization. Such a data structure is expected to help the model identify the controlling mechanism, relevant condition, or missing evidence behind a reported transport phenomenon. However, the reported results correspond to one selected corpus of 518 papers and one final SFT dataset of 8500 instruction–answer pairs. The 90.00% domain-oriented proportion combines mechanism explanation, method/simulation interpretation, structure–property reasoning, and parameter judgment; mechanism explanation alone accounts for 32.35%. These proportions describe the training design. No controlled variation of corpus size, instruction count, or category mixture was performed, and no matched generic-science SFT control was included. Consequently, the present results do not establish a scaling relationship, an optimal composition, or the separate causal contribution of mechanism-focused supervision.

The benchmark was constructed as the evaluation counterpart to this training objective. As shown in Figure 4, the 90-question benchmark was weighted toward mechanism-intensive and condition-dependent tasks. Mechanism explanation formed the largest module, followed by condition reasoning and concept distinction, while parameter judgment, literature-oriented reasoning, composition, structure–property relationship, and method-oriented reasoning were also included. This composition reflects the practical difficulty of desalination-related nanoconfinement, where the same macroscopic outcome, such as high-water flux or strong ion exclusion, may arise from different microscopic mechanisms depending on pore size, interlayer spacing, surface charge, wall chemistry, ion species, hydration structure, force-field selection, and simulation conditions.

The gold-point scoring scheme was therefore used to evaluate mechanism correctness rather than answer fluency alone. For example, a complete answer to why narrower nanochannels can increase ion exclusion should recover stronger confinement, greater ion dehydration requirements, and a higher pore-entry free-energy barrier, rather than relying only on a size-sieving explanation. Similarly, questions on fast water transport require the model to distinguish confined water ordering, water mobility, interfacial slip, wall–fluid friction, and residence or diffusion evidence. In this way, the benchmark penalizes common mechanism shortcuts and unsupported generalizations while rewarding concise recovery of physically meaningful structure–mechanism–transport relationships.

To isolate the contribution of domain fine-tuning, the main benchmark comparison in Section 3.2 was conducted without RAG for all evaluated models. The RAG module was analyzed separately as a source-traceable literature-grounding function and further examined through ablation analysis in later sections. This separation allows the quantitative benchmark results to reflect the effect of mechanism-oriented domain adaptation, while the RAG analysis evaluates whether retrieved literature evidence can support source-grounded interpretation.

### 3.2. Overall and Task-Level Benchmark Performance

The quantitative benchmark results demonstrate that NanoConfinementChat achieved the strongest overall performance among the three evaluated models. As summarized in Table 3 and Figure 5a, NanoConfinementChat reached an overall average score of 2.14, exceeding Qwen2.5-7B-Instruct (1.76) and the original Llama-3.1-8B-Instruct base model (1.46). Relative to the base model, this corresponds to an improvement of approximately 47.0%, indicating that domain-oriented supervised fine-tuning substantially enhanced the model’s ability to answer desalination-relevant nanoconfined-transport questions.

The improvement was even more evident in terms of scientific reliability. As shown in Figure 5b, NanoConfinementChat reduced the major scientific error rate to 2.22%, compared with 11.11% for the base model and 13.33% for Qwen2.5-7B-Instruct. This reduction is particularly important for mechanism-intensive membrane-science questions, because scientifically fluent but mechanistically incorrect answers can easily mislead interpretation of ion rejection, hydration effects, or interfacial transport phenomena. The low major-error rate suggests that NanoConfinementChat improved not only answer quality, but also the stability of core mechanism identification.

Task-level analysis further clarifies where the domain gains were concentrated. As shown in Figure 5c, NanoConfinementChat outperformed both baseline models across all four representative task modules. In the mechanism module, the average score increased from 0.92 for the base model and 1.56 for Qwen2.5-7B-Instruct to 1.96 for NanoConfinementChat, indicating a markedly improved ability to recover structure–mechanism–transport chains. In the literature-oriented module, NanoConfinementChat achieved 1.89, compared with 1.33 for the base model and 1.54 for Qwen2.5-7B-Instruct, suggesting better identification of evidence gaps and stronger interpretation of source-dependent claims. In the concept module, NanoConfinementChat reached 2.41, exceeding both the base model (2.00) and Qwen2.5-7B-Instruct (2.02), which indicates a clearer distinction between closely related but non-equivalent concepts such as dehydration barrier versus steric exclusion, or interfacial slip versus low friction. In the structure–property module, the model also showed the largest margin, scoring 2.17 compared with 1.33 for the base model and 1.67 for Qwen2.5-7B-Instruct, reflecting stronger reasoning from channel structure or surface chemistry to transport consequences.

Taken together, these results indicate that the benefit of NanoConfinementChat is not limited to a modest improvement in overall score. Rather, its main strength lies in more reliable mechanism reconstruction, more accurate concept discrimination, and stronger structure-to-transport interpretation, which are precisely the abilities required for mechanism-aware analysis of nanoconfined transport in ion-selective nanochannels. Although some uncertainty remains, as reflected by the 95% bootstrap confidence intervals in Figure 5, the descriptive trend is consistent across the overall benchmark and the representative task modules. As a supplementary reliability check, a stratified random subset of 11 benchmark questions was further reviewed by a domain expert using anonymized responses and the same gold-point and major-error criteria. This subset covered mechanism explanation, concept distinction, condition reasoning, method-oriented reasoning, literature/simulation interpretation, and structure–property relationship tasks. In this expert-reviewed subset, NanoConfinementChat achieved the highest mean score of 2.364, compared with 1.818 for the base model and 1.818 for Qwen2.5-7B-Instruct. It also produced no major scientific errors, whereas the base model and Qwen2.5-7B-Instruct produced one and two major errors, respectively. NanoConfinementChat obtained the highest or tied-highest score in all 11 reviewed questions. These results do not replace the full automatic benchmark, but they provide an additional human check supporting the observed reduction in mechanism-level errors. The detailed expert-review results are provided in the Supporting Information.

### 3.3. Representative Nanoscale Mass-Transfer Reasoning Cases

The practical meaning of the benchmark gains is illustrated by three representative nanoconfined transport cases in Figure 6. These cases were selected from mechanism-intensive and evidence-oriented benchmark questions because they reflect common interpretation challenges in desalination-related nanoconfined transport: explaining simultaneous fast water permeation and ion rejection, identifying why narrower channels can enhance ion exclusion, and judging whether a molecular dynamics study provides sufficient evidence for interfacial slip. The complete model outputs, predefined gold points, judge scores, and major-error labels for these cases are provided in the Supporting Information.

The first case concerns water/ion selectivity in subnanometer channels. A mechanism-resolved answer should recognize that water molecules may form single-file or ordered hydrogen-bonded chains under strong confinement, enabling concerted motion and efficient permeation. In contrast, hydrated ions usually face a much higher entry penalty because they must distort or partially shed their hydration shells before entering narrow pores. NanoConfinementChat recovered this coupled mechanism chain by linking ordered water transport to lower water permeation resistance and ion rejection to dehydration penalties and pore-entry free-energy barriers. This answer avoids a common shortcut in which fast water transport is explained mainly by hydrophilicity or pore size, without explaining why water and ions experience different transport barriers [10,11,16,32,33,34,35].

The second case concerns the effect of graphene oxide interlayer spacing on water/salt selectivity. A spacing-only explanation is incomplete because ion rejection in GO laminar membranes is governed by coupled geometric, hydration, and electrostatic effects. NanoConfinementChat connected reduced interlayer spacing to stronger confinement, increased ion dehydration penalty, higher pore-entry free-energy barriers, and charge-assisted exclusion. This interpretation is directly relevant to desalination membrane design because GO membranes with similar nominal spacing may show different selectivity depending on oxidation degree, surface charge, interlayer chemistry, hydration structure, and ion species. The case therefore shows that the model can move from a membrane design variable, interlayer spacing, to a physically grounded structure–mechanism–transport explanation [14,17,18,23,26,27,28,44].

The interfacial-slip case illustrates a concrete use for membrane researchers: identifying an underdetermined mechanism attribution before drawing conclusions from a reported high-water flux. The model response identifies velocity profiles, slip-length estimates, interfacial friction, and relevant simulation controls as evidence to examine alongside the flux observation. A researcher can use this output to organize checks of an existing MD study and distinguish a candidate explanation from a supported mechanistic conclusion. This is a qualitative illustration based on an existing benchmark case. No new trajectory, free-energy profile, characterization dataset, or experimental result was analyzed, and no previously unknown transport mechanism was established [32,33,34,35,36,37].

Together, these examples show that NanoConfinementChat is most effective in recovering compact structure–mechanism–transport chains and identifying missing mechanistic evidence. Rather than merely producing fluent descriptions, the model can help check whether a reported desalination performance trend is supported by plausible nanoscale mechanisms. The observed case-level behavior is consistent with the quantitative benchmark results in Section 3.2, where the largest gains appeared in mechanism-centered, literature-oriented, and structure–property tasks.

### 3.4. RAG-Supported Literature Grounding and Ablation Analysis

In addition to parameterized model responses, NanoConfinementChat integrates a retrieval-augmented generation module to support source-traceable literature-grounded analysis [40,41,42,43]. The benchmark comparison in Section 3.2 was conducted without RAG to isolate the effect of domain fine-tuning, whereas RAG was evaluated separately as a source-grounding component. In this mode, a user query is embedded and matched to evidence chunks from the curated corpus. The retrieved chunks retain article titles and page numbers and are inserted into a source-grounded prompt to guide response generation. This design is useful for nanoconfined transport analysis because mechanistic conclusions often depend on source-specific conditions, including pore size, interlayer spacing, ion type, solution concentration, surface charge, force-field parameters, thermostat choice, and boundary conditions.

Figure 7 illustrates the RAG-supported workflow through a representative literature-grounded case. The query asks whether fast water transport in carbon nanotubes is mechanistically underexplained when interfacial friction or slip is not discussed. The retrieved chunks provide source metadata and relevant transport evidence, and the generated response identifies interfacial friction or slip as the missing mechanistic explanation. The example shows that RAG can support source-traceable evidence checking by linking a generated judgment to retrieved literature context. In this case, high water flux alone is insufficient to prove interfacial slip; more direct evidence should include velocity profiles near the wall, slip-length estimation, water-wall friction analysis, density layering or water ordering, and controls for force field, driving force, thermostat, and boundary conditions.

To further examine the role of retrieval grounding, we performed an ablation analysis on an evidence-dependent subset excluding parameter-only questions. This subset contained 18 questions from the literature-oriented reasoning, method-oriented reasoning, and structure–property relationship modules. Four settings were compared: the base model, the base model with RAG, the SFT model without RAG, and the SFT model with RAG. This subset analysis was designed to diagnose how retrieval grounding interacts with domain adaptation under evidence-dependent questions, rather than to replace the full 90-question benchmark comparison.

The results are summarized in Table 4. Applying RAG directly to the base model reduced the average score from 2.278 to 1.278 and increased the major scientific error rate from 2/18 (11.11%) to 6/18 (33.33%). This result indicates that retrieval alone was not sufficient for mechanism-aware interpretation. Retrieved chunks may provide useful source context, but an unadapted model may overfocus on partially aligned evidence, introduce overly source-specific details, or fail to integrate retrieved snippets into a correct mechanism-level judgment. For the domain-adapted model, adding RAG increased the average score from 1.333 to 1.500, suggesting that retrieved evidence helped recover additional gold-point information in some evidence-dependent questions. However, the major scientific error rate also increased from 2/18 (11.11%) to 4/18 (22.22%). Therefore, the current RAG module should not be interpreted as a consistent answer-accuracy booster.

Taken together, the ablation results indicate that domain fine-tuning and RAG play different roles in the current system. The main benchmark improvement reported in Section 3.2 was obtained without RAG and therefore primarily reflects mechanism-oriented domain fine-tuning. RAG complements this capability by providing source traceability, page-level evidence, and literature-grounded context for inspecting mechanistic claims. Its value is most evident when the goal is to check whether a claim is supported, underexplained, or dependent on source-specific evidence. At the same time, its effectiveness depends on retrieval relevance, chunk quality, and prompt design.

The current RAG module is intended to support auditability and provenance by retaining article titles and page numbers and making the retrieved text available for inspection. The ablation does not establish conditions under which retrieval reliably improves mechanistic accuracy: adding RAG to the SFT model increased the mean score from 1.333 to 1.500 while increasing major scientific errors from 2/18 to 4/18. Source traceability does not establish that a cited passage supports every generated claim or matches the conditions of the question. Citation support, condition matching, and evidence-integration errors require separate assessment.

### 3.5. Implications, Limitations, and Future Optimization

The development of NanoConfinementChat demonstrates the potential of domain-specific adaptation to support mechanism-aware interpretation of water and ion transport in ion-selective nanochannels. This capability is relevant to membrane research because macroscopic transport performance often reflects the combined effects of several microscopic mechanisms. The present findings should nevertheless be interpreted within the scope of the evaluated models and benchmark. The full benchmark compares NanoConfinementChat with two general-purpose models under the reported direct-answer setting. It does not include another domain-specific generative scientific model or a dedicated long-context baseline supplied with matched literature evidence. The 18-question base-model RAG comparison does not resolve this limitation. In addition, no matched control trained on generic scientific instruction data was evaluated. The observed gains therefore cannot establish superiority over these alternatives or isolate the contribution of mechanism-focused supervision from other effects of additional fine-tuning.

One important implication of this work is that NanoConfinementChat can help reduce common size-sieving shortcuts in the interpretation of nanoconfined transport. The benchmark results and representative cases suggest that mechanism-oriented fine-tuning helps the model incorporate ion dehydration penalties, electrostatic effects, and pore-entry free-energy barriers into explanations of ion exclusion. This capability is useful for examining whether a reported transport trend has an adequate mechanistic explanation. However, incomplete gold-point coverage in mechanism explanation and literature-oriented reasoning, together with the two responses containing major scientific errors in the full benchmark, shows that the model still requires verification. Questions involving competing mechanisms, quantitative parameter judgments, or transfer of conclusions between material systems warrant particular scrutiny, although their individual failure frequencies have not been established. When pore size, ion species, surface chemistry, solution concentration, or simulation conditions are incompletely specified, a plausible explanation may be generalized beyond its supporting conditions. Similarly, identifying a possible mechanism does not establish that the available evidence demonstrates its contribution. Such questions require explicit assumptions, acknowledgment of missing information, and careful checking against the original literature.

The ablation analysis further clarifies the relationship between domain fine-tuning and retrieval-augmented generation. In the reported subset, applying RAG to the base model reduced answer quality, while adding RAG to the SFT model produced a modest increase in mean score alongside an increase in the major scientific error rate. These mixed results do not establish a consistent accuracy benefit from retrieval or show that fine-tuning reliably resolves evidence-integration errors. Partially relevant passages or differences between the conditions described in the retrieved evidence and those specified in the question may contribute to incorrect interpretations, but these possibilities require direct examination of the retrieved passages and responses. In nanoconfined transport, evidence concerning slip length, hydration structure, or PMF profiles must be interpreted in relation to pore geometry, surface charge, force-field choice, driving force, and boundary conditions. The current RAG module therefore provides a means of tracing and inspecting supporting sources, while the scientific validity of the resulting interpretation still requires verification. Future evaluation should assess whether cited passages support the associated claims and whether their conditions match the question.

Several limitations remain. The evaluation remains an initial assessment on a 90-question internal benchmark. The supplementary 11-question expert review is too limited to establish mechanism-level accuracy across the field. The benchmark has not been expanded, and a substantial external expert validation has not been performed. Broader question coverage and independent expert assessment remain necessary before drawing conclusions about performance on unfamiliar membrane systems or conditions. Although a stratified subset was manually reviewed by a domain expert, the full 90-question benchmark relies on automatic judging, which may not consistently resolve subtle or condition-dependent mechanistic claims. Expanded expert review and systematic analysis of disagreements with the automatic judge would strengthen the evaluation and help characterize residual failure modes. The current system is also text-centered, whereas important evidence in nanoconfined-transport literature is frequently presented in figures, tables, and simulation snapshots, including PMF profiles, radial distribution functions, velocity profiles, ion-density distributions, and membrane morphology images. Multimodal retrieval and evidence assessment could extend the range of information available for mechanism checking, although any resulting improvement would require validation. Future optimization could also connect NanoConfinementChat with molecular dynamics simulators, curated transport databases, and experimental membrane datasets. Shared literature sources between training and evaluation introduce a potential risk of data leakage and memorization. The present evaluation cannot determine how much of the observed improvement reflects generalizable mechanism reasoning. Leakage-controlled validation using source-paper-disjoint splits, cross-dataset overlap audits, and independently collected literature remains future work. These developments, together with broader baseline comparisons and targeted assessment of difficult questions, could support more reliable concept verification and hypothesis generation in membrane science.

## 4. Conclusions

This work developed a domain-specific large language model, NanoConfinementChat, tailored for the mechanism-aware reasoning of nanoscale mass transfer in ion-selective membrane nanochannels. By integrating a curated nanoconfined-transport corpus, mechanism-oriented instruction data, LoRA-based supervised fine-tuning, and a customized gold-point benchmark, the study successfully adapted a lightweight open-source model to address complex, mechanism-intensive transport problems in confined geometries. Evaluated on a 90-question benchmark without RAG, NanoConfinementChat achieved the highest overall score of 2.144 and reduced the major scientific error rate to 2.22%. The model demonstrated significant advantages in recovering intricate structure–mechanism–transport reasoning chains involving ordered water transport in subnanometer channels, dehydration-driven ion exclusion, pore-entry free-energy barriers, electrostatic exclusion, graphene oxide interlayer-spacing dynamics, and evidentiary requirements for interfacial slip. While these results indicate that targeted domain datasets and lightweight fine-tuning fundamentally improve the reliability of mechanism-level interpretation, several limitations remain to be addressed, including the reliance on automatic judge-based scoring and the constraints of a text-centered RAG module that cannot directly interpret non-textual evidence (e.g., potential of mean force curves or molecular simulation snapshots). By incorporating leakage-controlled benchmark construction and multimodal evidence reasoning in future work, NanoConfinementChat will continue to provide a reusable and extensible framework for the evidence-grounded reasoning of nanoconfined transport, ultimately supporting more reliable analysis of mass flux, transport barriers, and separation selectivities in advanced membrane nanochannels.

## Figures and Tables

**Figure 1:**
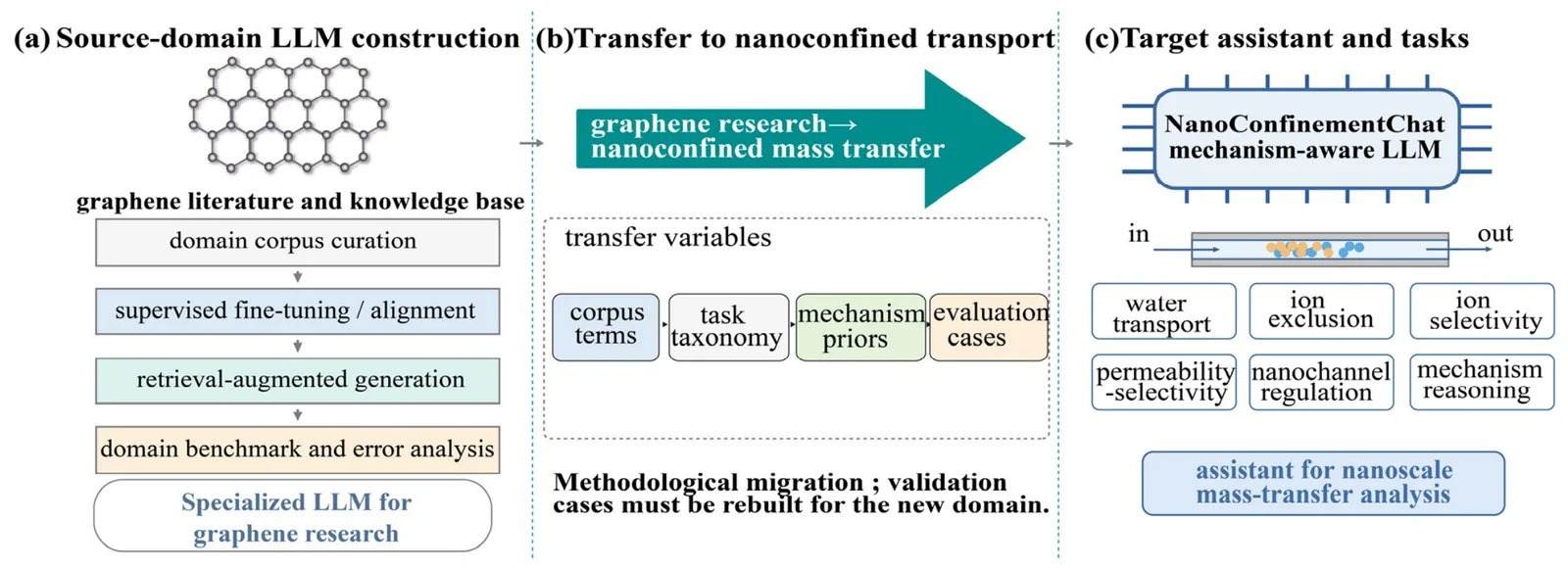
Conceptual framework and domain adaptation strategy of NanoConfinementChat. (**a**) Source-domain scientific LLM construction. (**b**) Methodological transfer from graphene research to nanoconfined mass-transfer reasoning by rebuilding domain-specific variables and validation cases. (**c**) Target assistant designed for mechanism-aware analysis of water and ion transport in ion-selective membrane nanochannels.

**Figure 2:**
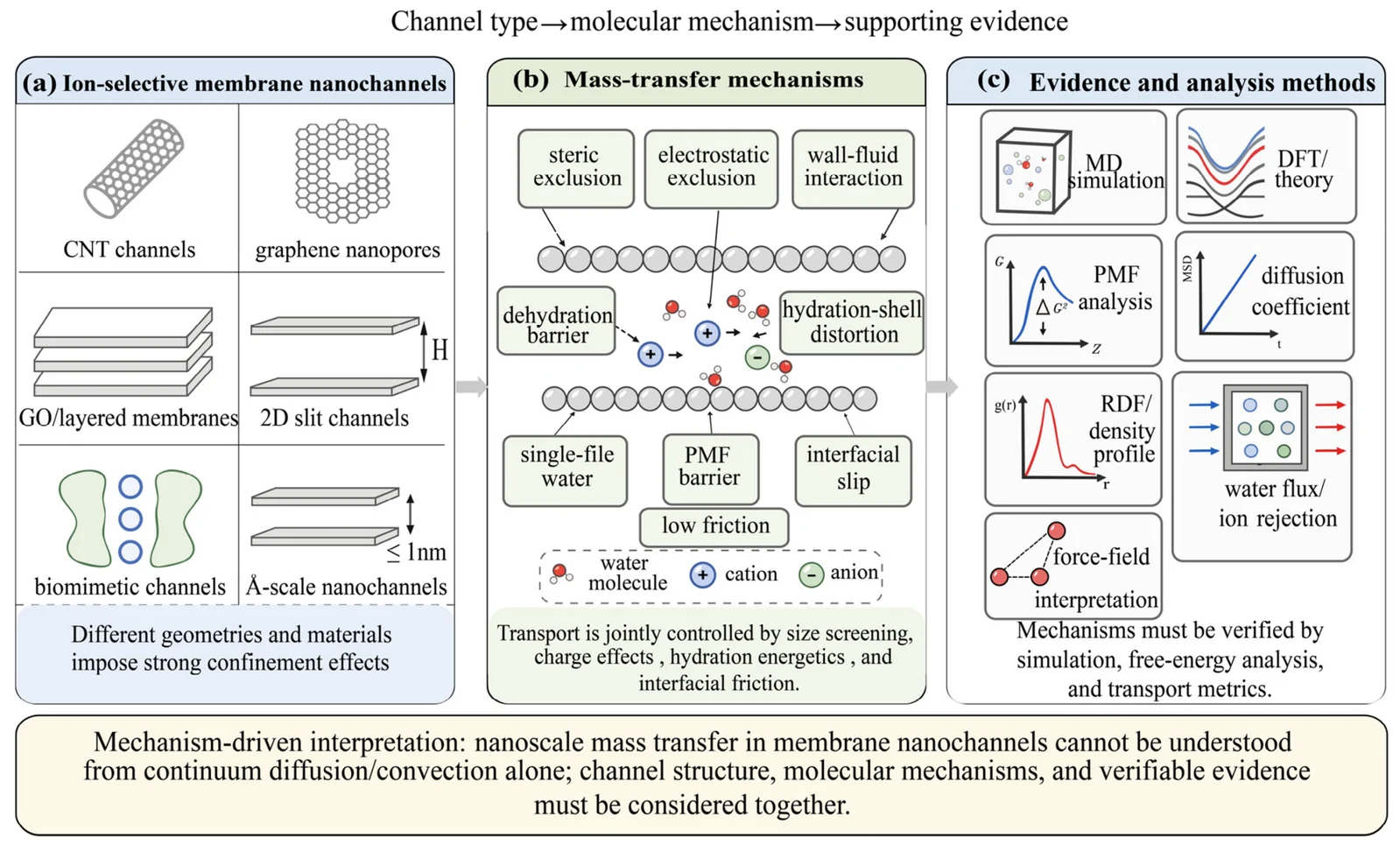
Structured knowledge organization for nanoscale mass transfer in membrane nanochannels. (**a**) Representative ion selective membrane nanochannels covered by the curated corpus. (**b**) Key molecular mechanisms governing confined water and ion transport. (**c**) Evidence and analysis methods used to support mechanism interpretation. The framework links nanochannel systems, transport mechanisms, and verifiable evidence for instruction generation, retrieval grounding, and benchmark design.

**Figure 3:**
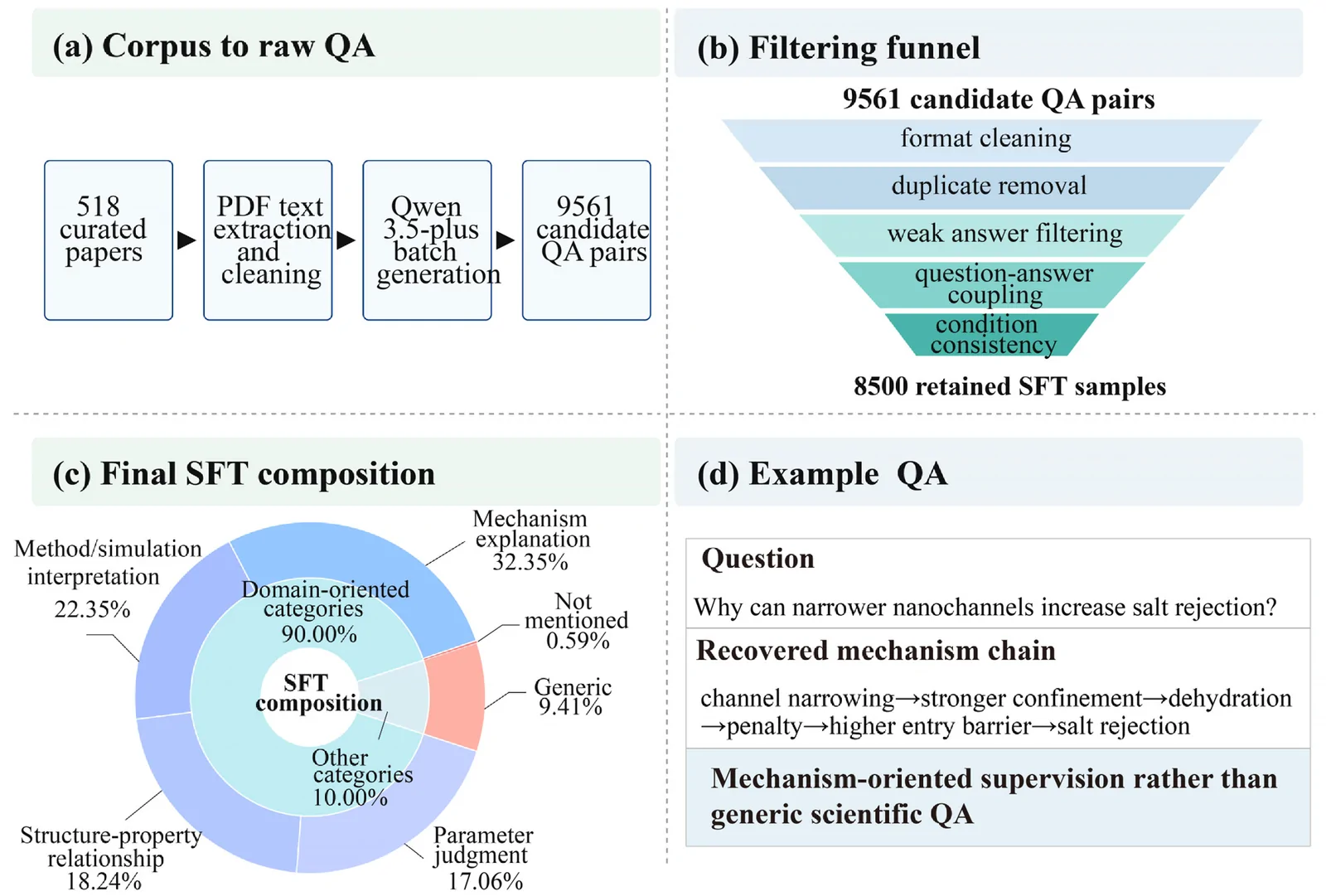
Construction and composition of the supervised fine-tuning dataset for NanoConfinementChat. (**a**) Corpus to raw question–answer (QA) pair generation from curated papers using text extraction, cleaning, and batch question–answer generation. (**b**) Filtering funnel used to obtain high-quality SFT samples through format, duplication, answer quality, question–answer matching, and condition consistency checks. (**c**) Final SFT composition showing that domain-oriented categories account for 90.00% of the dataset. (**d**) Representative mechanism-oriented QA example illustrating supervision focused on causal mechanism chain recovery.

**Figure 4:**
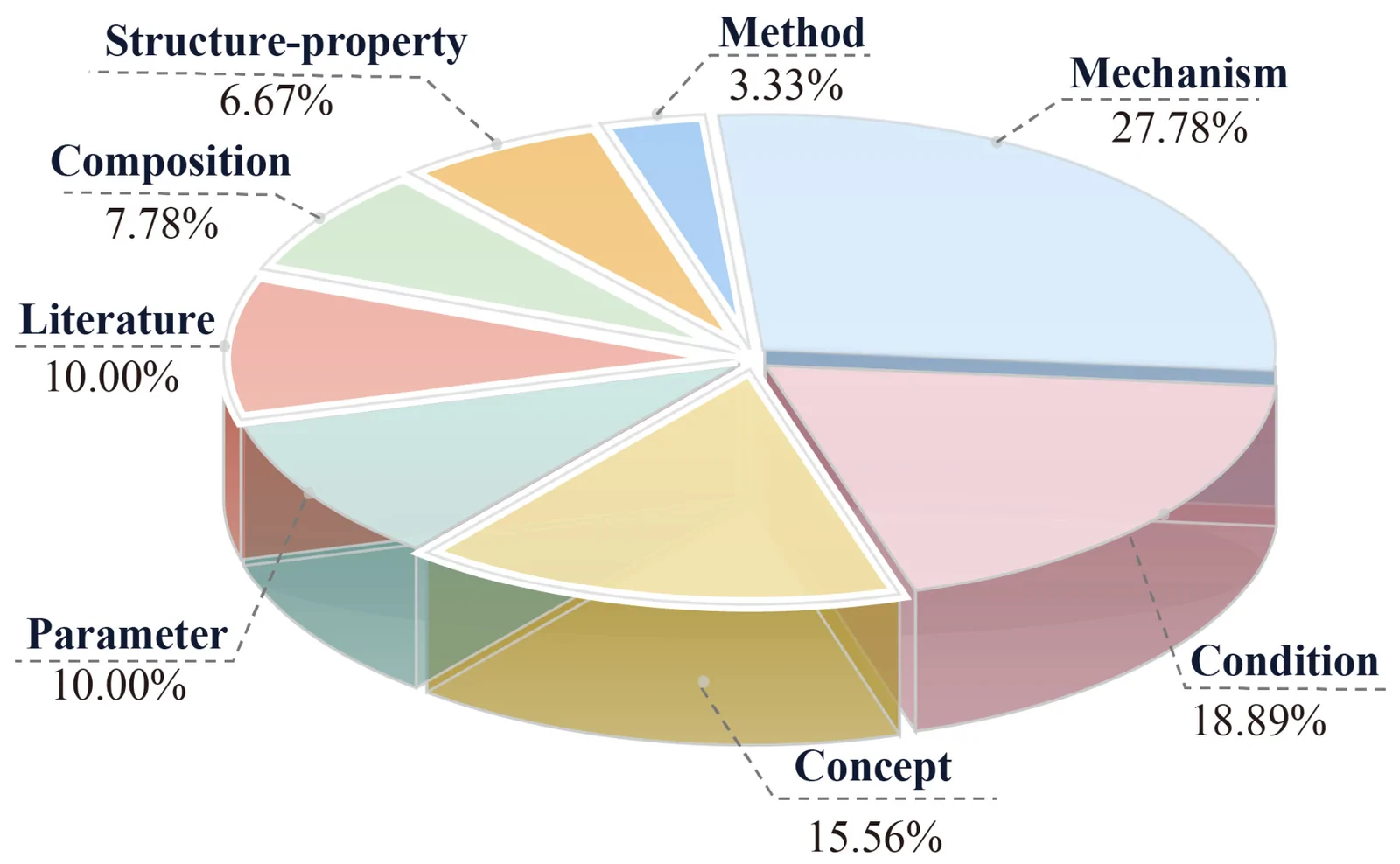
Task distribution of the 90-question benchmark across major evaluation categories.

**Figure 5:**
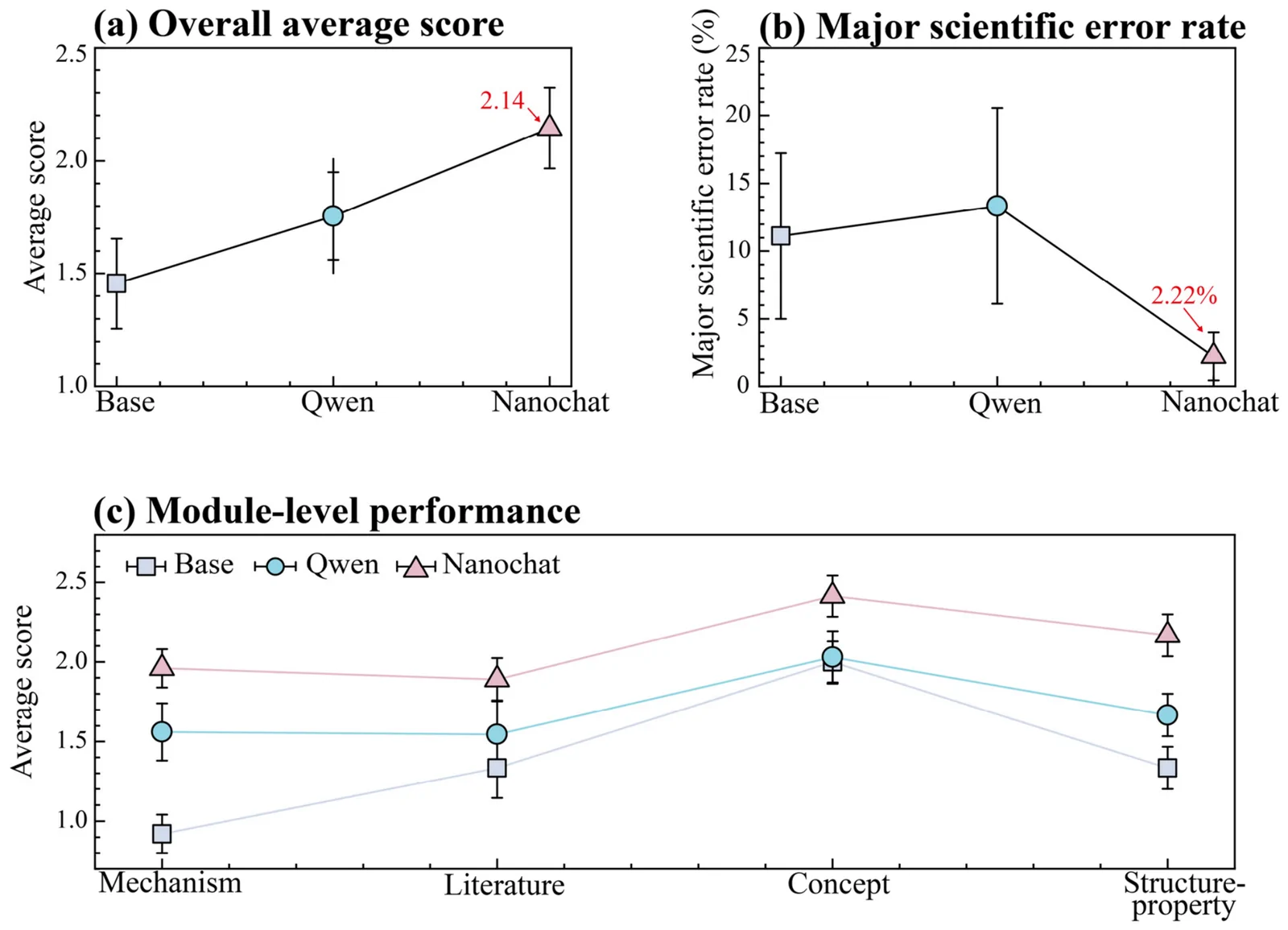
Overall and task-level benchmark performance of NanoConfinementChat and baseline models. The original Llama 3.1 8B Instruct model is denoted as Base, Qwen2.5 7B Instruct as Qwen, and NanoConfinementChat as NanoChat. Error bars represent 95% bootstrap confidence intervals. (**a**) Overall average score on the 90-question benchmark. (**b**) Major scientific error rate. (**c**) Module-level performance in four representative task categories, showing the domain adaptation effect of NanoConfinementChat.

**Figure 6:**
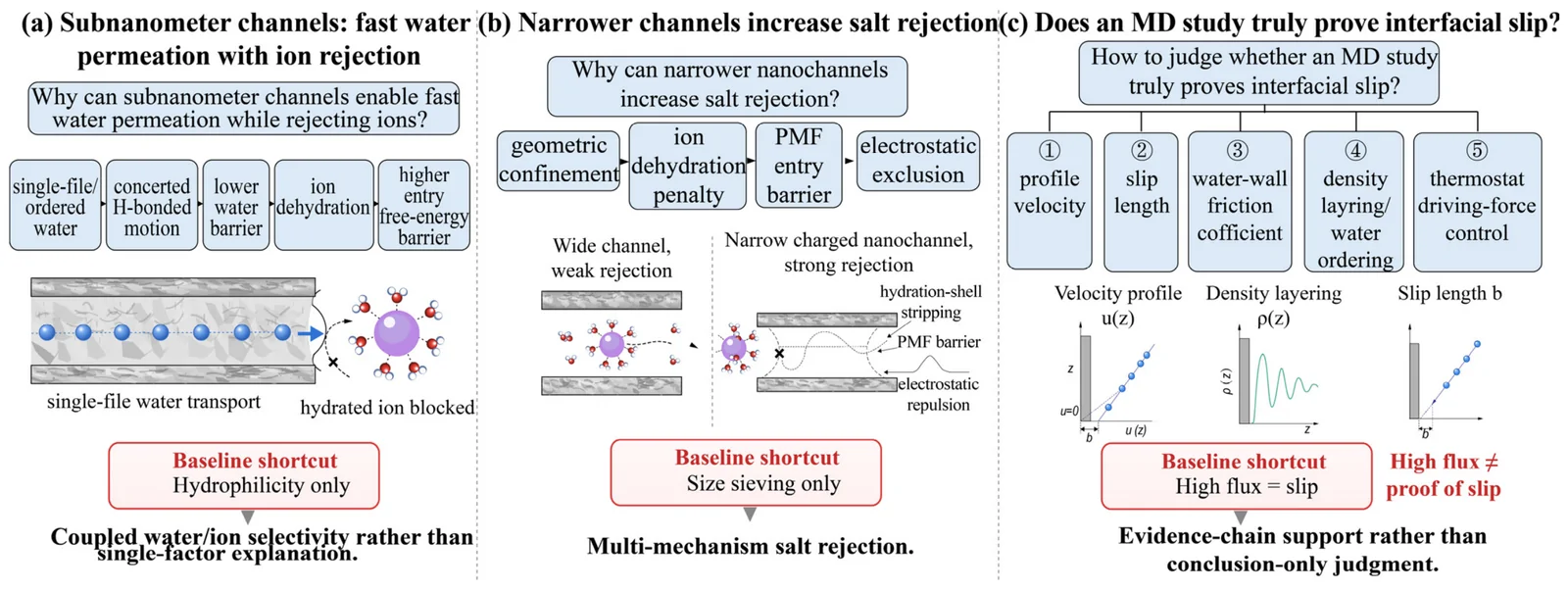
Representative mechanism reasoning cases for NanoConfinementChat. (**a**) Subnanometer channel case showing coupled fast water permeation and ion rejection. (**b**) Narrow channel case showing multi-mechanism enhancement of salt rejection. (**c**) Interfacial slip case showing the evidence required to judge whether MD results truly support slip. These cases illustrate mechanism chain recovery and evidence-based reasoning beyond common single factor explanations.

**Figure 7:**
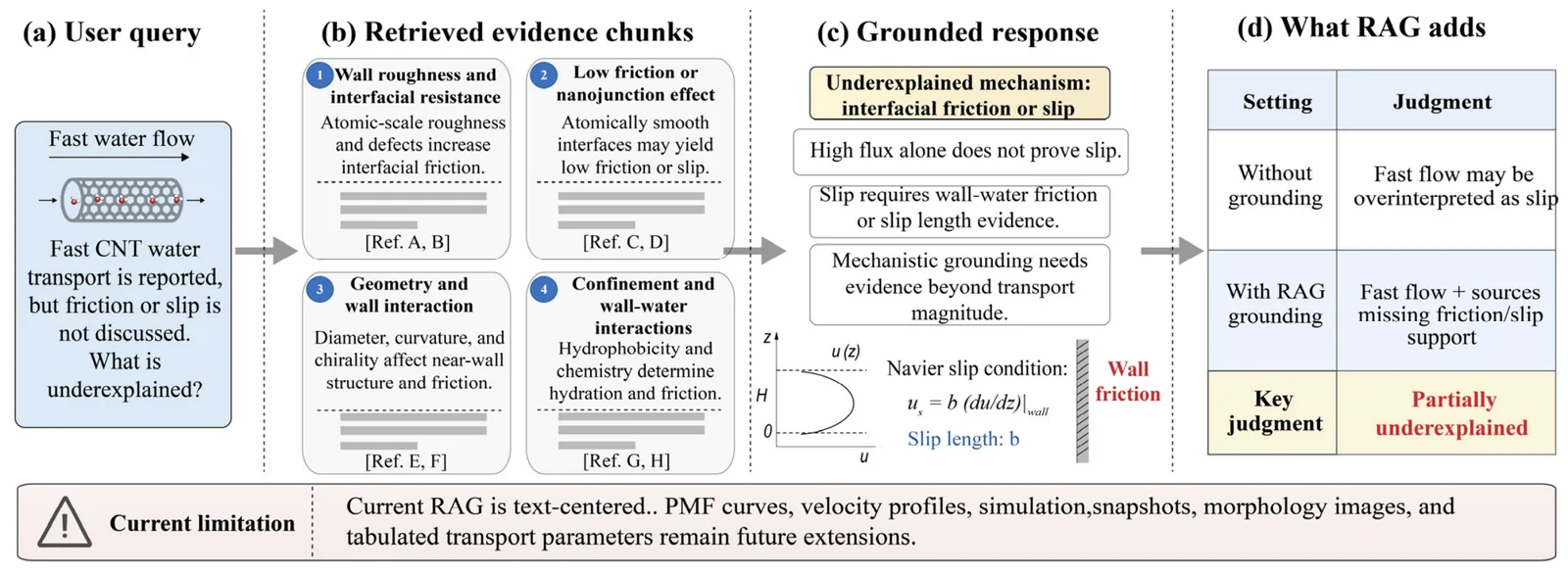
Retrieval-augmented generation workflow for source-traceable mechanism analysis in NanoConfinementChat. (**a**) User query on the missing friction or slip explanation in fast CNT water transport. (**b**) Retrieved literature evidence related to CNT wall interaction, interfacial resistance, geometry effects, and confinement effects. (**c**) Grounded response identifying interfacial friction or slip as the underexplained mechanism. (**d**) RAG-supported evidence checking that distinguishes unsupported fast flow interpretation from literature-grounded mechanistic judgment. The limitation note highlights the current text-centered nature of the RAG module.

**Table 1:** Domain-specific adaptation strategy for NanoConfinementChat.

Component	General Domain-Specific LLM Workflow	NanoConfinementChat Implementation
Domain	Graphene research	Nanoconfined transport in ion-selective nanochannels
Core knowledge	Synthesis, characterization, properties, and applications	Water/ion transport, permeability, selectivity, ion exclusion, and transport mechanisms
Corpus	Graphene-related literature and domain materials	Nanoconfined-transport papers linked to ion-selective membrane nanochannels
Training target	Graphene-specific knowledge retrieval and experimental assistance	Mechanism explanation, condition reasoning, structure–transport relationships, and parameter-aware interpretation
RAG function	Literature-grounded graphene question-answering	Evidence-supported interpretation of nanoconfined transport mechanisms
Benchmark	GrapheneBench for graphene research	Gold-point benchmark for mechanism correctness in nanoconfined transport

**Table 2:** Main configuration used for LoRA-based supervised fine-tuning of NanoConfinementChat.

Parameter	Setting
Base model	Llama-3.1-8B-Instruct
Training framework	LLaMA-Factory
Training stage	supervised fine-tuning
Fine-tuning strategy	LoRA
LoRA target	all
LoRA rank/alpha/dropout	8/16/0.0
Template	llama3
Maximum sequence length	2048
Learning rate	5 × 10^−5^
Epochs	3
Per-device batch size	2
Gradient accumulation steps	4
Effective batch size	8
Scheduler	cosine
Warmup ratio	0.1
Precision	FP16
Optimizer	paged AdamW 8-bit
Gradient checkpointing	enabled
Trainable parameters	20,971,520
Trainable parameter ratio	0.2605%
GPU	NVIDIA A10 × 1
Training time	1 h 11 min 54 s
Final training loss	1.4692

**Table 3:** Overall benchmark performance of NanoConfinementChat and baseline models.

Model	Overall Score, Mean [95% CI]	Relative Gain vs. Base (%)	Major Error Rate [95% CI]	Rank
Llama-3.1-8B-Instruct base	1.4556 [1.2556–1.6556]	Reference	11.11% [5.00–17.22%]	3
Qwen2.5-7B-Instruct	1.7556 [1.5611–1.9500]	+20.6	13.33% [6.11–20.56%]	2
NanoConfinementChat	2.1444 [1.9667–2.3222]	+47.3	2.22% [0.44–4.00%]	1

Note: Scores were calculated using the benchmark evaluation protocol described in Section 2.6. The major scientific error rate denotes the fraction of benchmark questions for which the answer contained a clear scientific error, mechanism reversal, or serious concept confusion.

**Table 4:** Ablation analysis of RAG on the evidence-dependent benchmark subset.

Setting	Fine-Tuning	RAG	n	Overall Score	Major Scientific Error, n/N (%)
Base only	No	No	18	2.278	2/18 (11.11%)
Base + RAG	No	Yes	18	1.278	6/18 (33.33%)
SFT only	Yes	No	18	1.333	2/18 (11.11%)
SFT + RAG	Yes	Yes	18	1.500	4/18 (22.22%)

Note: This subset was used to diagnose retrieval-grounding behavior on evidence-dependent questions and was not intended as a replacement for the full 90-question benchmark ranking in Section 3.2.

## Data Availability

The complete 90-question benchmark and predefined gold points (newest test_90.jsonl), automatic scoring protocol (Table S7), detailed model responses and evaluation records (judge_detail_scores.json; Table S9), representative case records (case_answer_summary.csv; Table S11), and summary of the 11-question expert review (Table S12) are provided in the Supporting Information accompanying this manuscript.

## Supplementary Materials

The following supporting information can be downloaded at: https://www.mdpi.com/article/10.3390/membranes16100319/s1, Table S1. Full OpenAlex query terms and exclusion terms used for literature retrieval; Table S2. Domain taxonomy used for corpus organization; Table S3. Prompt design for instruction–answer generation (including the suggested prompt template for SI information); Table S4. Data filtering rules for QA cleaning and logic-consistency screening (including example KEEP/DISCARD decision format); Table S5. Detailed LoRA training and inference configuration; Table S6. RAG implementation details; Table S7. DeepSeek judge prompt, JSON output format, and scoring rules; Table S8. Benchmark module distribution and complete 90-question benchmark; Table S9. Per-question model scores and major-error labels; Table S10. Ablation analysis of RAG on the evidence-dependent benchmark subset; Table S11. Representative case responses and gold-point evaluation; Table S12. Summary of expert manual review.

## Abbreviations

The following abbreviations are used in this manuscript:

CNT	carbon nanotube
GO	graphene oxide
MD	molecular dynamics
DFT	density functional theory
PMF	potential of mean force
RDF	radial distribution function
MSD	mean squared displacement
2D	two-dimensional
QA	question–answer
SFT	supervised fine-tuning
PDF	Portable Document Format
