# Supplementary material for: NanoConfinementChat: A Domain-Specific Large Language Model for Mechanistic Interpretation of Nanoconfined Water and Ion Transport in Membrane Nanochannels
Source: Membranes (Basel). 2026 Sep 26;16(10):319. doi: 10.3390/membranes16100319 (PMC13707350; doi:10.3390/membranes16100319)
Supplement: Supplementary file 1 [file membranes-16-00319-s001.zip › Supporting Information.pdf]

Supporting information

The Supporting Information provides detailed methodological materials to support reproducibility, including the full literature search terms, domain taxonomy, prompt design, filtering rules, LoRA training configuration, RAG implementation parameters, DeepSeek judge scoring protocol, complete benchmark composition, and per-question model scores. These details complement the main Methods section without overloading the main text with implementation-specific details. Furthermore, the complete 90-question benchmark dataset, detailed model responses for representative cases, and automated judge scoring logs are provided as separate machine-readable supplementary files (json, jsonl, and csv).

List of Tables

Table S1. Full OpenAlex query terms and exclusion terms used for literature retrieval.

Table S2. Domain taxonomy used for corpus organization.

Table S3. Prompt design for instruction–answer generation (including the suggested prompt template for SI information).

Table S4. Data filtering rules for QA cleaning and logic-consistency screening (including example KEEP/DISCARD decision format).

Table S5. Detailed LoRA training and inference configuration.

Table S6. RAG implementation details.

Table S7. DeepSeek judge prompt, JSON output format, and scoring rules.

Table S8. Benchmark module distribution and complete 90-question benchmark.

Table S9. Per-question model scores and major-error labels.

Table S10. Ablation analysis of RAG on the evidence-dependent benchmark subset.

Table S11. Representative case responses and gold-point evaluation.

Table S12. Summary of expert manual review.

Table S1. Full OpenAlex query terms and exclusion terms used for literature retrieval.

Category	Query terms
Database	OpenAlex API
Search date	11 April 2026
Publication years	2015–2025
Ranking criterion	cited_by_count:desc
DOI requirement	Only records with DOI were retained
Nanoconfined-transport terms	nanoconfined transport; nano-confined transport; transport under nanoconfinement; nanoconfinement effect; confined transport; confined water; confined ion transport; confined fluid transport; subnanometer channel; sub-nanometer channel; angstrom-scale channel; nanopore transport; nanochannel transport; single-file water; interlayer nanochannel; 2D nanochannel
Transport descriptors	water transport; ion transport; molecular transport; permeation; diffusion; flow; selectivity; water permeation; ion permeation; osmotic transport
Mechanism and method terms	molecular dynamics; simulation; theoretical; theory; modeling; free energy; energy barrier; hydrophobicity; hydration; hydrogen-bond network; friction; slip; slip length; transport mechanism; structure-property relationship
Material-system terms	aquaporin; water channel protein; biomimetic water channel; carbon nanotube; CNT; graphene; graphene oxide; membrane; nanopore; nanochannel
Exclusion terms	photocatalysis; battery; supercapacitor; sensor; electrode; solar cell; therapy; cancer; imaging; robot; actuator; alloy
Initial candidate scale	Approximately 1000 candidate papers
Final corpus size	518 papers
Category	Query terms

Note: The search was performed using the OpenAlex Works endpoint, with

journal-source information obtained through the OpenAlex Sources endpoint.

Table S2. Domain taxonomy used for corpus organization.

Taxonomy level	Labels
Material-system labels	Carbon nanotube / CNT channels; graphene channels; graphene oxide / GO membranes; graphene or GO slit channels; 2D layered membranes; interlayer nanochannels; nanopores; nanochannels; subnanometer channels; Å-scale channels; aquaporins; biomimetic water channels; water channel proteins
Mechanism labels	Dehydration barrier; hydration-shell distortion; steric exclusion; interfacial slip; low interfacial friction; wall–fluid interaction; wall–ion interaction; wall–water interaction; electrostatic exclusion; electrostatic screening under confinement; hydrogen-bond network rearrangement; single-file water transport; ordered water chain; free-energy barrier; PMF / free-energy profile; structure–property relationship; water–ion transport decoupling; entry barrier / partitioning barrier
Method labels	Molecular dynamics simulation; atomistic simulation; DFT / first-principles calculation; theoretical modeling; continuum-model comparison; free-energy calculation; PMF analysis; diffusion coefficient analysis; water flux / permeability analysis; ion rejection analysis; force-field-dependent simulation interpretation; structure–property analysis; literature-based mechanism interpretation

Table S3. Prompt design for instruction–answer generation.

Item	Description
QA generation model	qwen3.5-plus
Inference interface	Qwen Batch API
Input format	Cleaned full-text paper content
Output format	JSON-formatted instruction–answer pairs

Target QA types	Mechanism-oriented QA; structure–property QA; method/simulation QA; concept-discrimination QA; literature-interpretation QA
Prompt objective	Generate domain-specific instruction–answer pairs for nanoconfined transport rather than generic paper summaries
Grounding requirement	Answers should remain close to the source text and avoid unsupported extrapolation
Scientific emphasis	Physical mechanisms; simulation interpretation; key transport parameters; structure–property relationships; literature-supported reasoning
Excluded response style	Generic summaries; vague explanations; unsupported claims; broad membrane-science descriptions without nanoconfined-transport mechanisms

Suggested prompt template for SI

Prompt component	Content
Role	You are an expert in nanoconfined water and ion transport, desalination membranes, molecular simulation, and transport mechanisms.
Task	Read the provided scientific text and generate high-quality instruction–answer pairs related to nanoconfined transport.
Required question types	Generate questions involving mechanism explanation, structure–property relationship, method/simulation interpretation, concept discrimination, and literature-based mechanism analysis.
Answer requirements	Answers must be grounded in the provided text, scientifically precise, and focused on physical mechanisms, simulation evidence, key parameters, and structure–transport relationships.
Avoid	Do not generate generic reading-comprehension questions, unsupported claims, or broad membrane-performance summaries without mechanism discussion.
Output	Return a JSON object or JSON list

	containing instruction–answer pairs.

Table S4. Data filtering rules for QA cleaning and logic-consistency screening.

Filtering stage	Rule
Basic parsing	Parse nested API outputs and extract valid generated content
JSON validation	Parse JSON-formatted QA pairs from generated content
Empty-field filtering	Remove samples with empty question or empty answer
Short-answer filtering	Remove extremely short answers, e.g., answers with insufficient semantic content
Duplicate filtering	Remove repeated or near-identical questions
Format conversion	Convert valid QA pairs into LLaMA-Factory format: instruction, input, output
Logic consistency	Check whether the answer directly addresses the question
Object consistency	Check whether question and answer refer to the same material system, mechanism, parameter, condition, or conclusion
Mechanism consistency	Check whether the answer explains the mechanism requested by the question
Off-topic answer	Answer does not address the question or only shares superficial keyword overlap
Missing core information	Answer lacks the essential mechanism, parameter, or conclusion required by the question
Semantic mismatch	Question and answer discuss different objects, mechanisms, or conditions
Overly generic answer	Answer is vague, shallow, or broadly scientific without domain-specific content

Example KEEP/DISCARD decision format

Example type	Question	Answer behavior	Decision
Mechanism matched	Why can narrower nanochannels enhance salt rejection?	Explains stronger confinement, ion dehydration requirement, and higher entry free-energy barrier	KEEP

Concept mismatch	What distinguishes dehydration barrier from steric exclusion?	Only states that smaller pores reject ions without discussing dehydration	DISCARD
Off-topic answer	How does interfacial slip affect water transport?	Discusses general membrane fouling without slip or wall–water friction	DISCARD
Overly generic	What controls ion transport in nanoconfinement?	States "many factors affect transport" without naming mechanisms or parameters	DISCARD

Table S5. Detailed LoRA training and inference configuration.

Parameter	Setting
Base model	Llama-3.1-8B-Instruct
Training framework	LLaMA-Factory
Training stage	Supervised fine-tuning
Fine-tuning strategy	LoRA
LoRA target	all
LoRA rank	8
LoRA alpha	16
LoRA dropout	0.0
Prompt template	llama3
Maximum sequence length	2048
Learning rate	5×10^{-5}
Epochs	3
Per-device batch size	2
Gradient accumulation steps	4
Effective batch size	8
Scheduler	cosine
Warmup ratio	0.1
Precision	FP16
Optimizer	paged AdamW 8-bit
Gradient checkpointing	enabled
Trainable parameters	20,971,520
Total parameters	8,051,232,768
Trainable parameter ratio	0.2605%
GPU	NVIDIA A10 × 1
Training device	single GPU, cuda:0

Distributed training	False
Training time	approximately 1 h 11 min 54 s

Table S6. RAG implementation details.

Component	Setting
RAG corpus	Curated PDF corpus from 518 nanoconfined-transport papers
Metadata fields	paper_id; article_title; pdf_name; source path / OSS path; page_number
Chunking strategy	Page-level parsing followed by overlapping text chunking
Chunk size	800 characters
Chunk overlap	150 characters
Chunk record format	JSONL
Chunk-level metadata	paper_id; article_title; pdf_name; source path / OSS path; page_number
Embedding model	DashScope text-embedding-v4
Vector database	ChromaDB
Vector database mode	Persistent local vector store
Retrieval similarity	Cosine similarity
Notebook retrieval top-k	4
Lightweight web-interface top-k	1
RAG generation model	Merged fine-tuned Llama-3.1 SFT model
Source-grounded prompt requirement	Use only information supported by retrieved snippets
Uncertainty handling	State uncertainty when retrieved evidence is incomplete
Anti-fabrication instruction	Do not fabricate papers, page numbers, mechanisms, or conclusions
Source citation format	[Source: Article Title, Page X]
Notebook generation setting	max_new_tokens = 220; temperature = 0.2; top_p = 0.9; repetition_penalty = 1.15
Lightweight web generation setting	max_new_tokens = 80; do_sample = False; repetition_penalty = 1.1
Web interface	Flask-based demo interface

Table S7. DeepSeek judge prompt, JSON output format, and scoring rules.

Item	Description
Judge model	deepseek-chat
API provider	DeepSeek API
Maximum judge output length	220 tokens
Evaluated models	NanoConfinementChat; Llama-3.1-8B-

	Instruct base; Qwen2.5-7B-Instruct
Evaluation unit	One model answer to one benchmark question
Main scoring criterion	Whether the answer covers predefined gold points
Gold-point matching	Scientifically equivalent paraphrases, near-synonymous expressions, and clearly implied coverage were accepted
Verbosity rule	Long answers were not rewarded merely for length
Conciseness rule	Short answers were not penalized if core scientific points were correctly covered
Major-error criterion	Assigned only when the answer contained clear scientific error, conceptual confusion, mechanism reversal, severe off-topic reasoning, or misleading core judgment
Output format	JSON
Required JSON fields	gold_point_1_hit; gold_point_2_hit; gold_point_3_hit; major_error; rationale
Raw score	gold_point_1_hit + gold_point_2_hit + gold_point_3_hit - major_error
Final score	max(raw_score, 0)
Manual review	No additional manual review was conducted in the current evaluation round

Suggested JSON output format

{

"gold_point_1_hit": 1,

"gold_point_2_hit": 1,

"gold_point_3_hit": 1,

"major_error": 0,

"rationale": "The answer covers the dehydration requirement and entry barrier but does not clearly mention geometric confinement."

}

Table S8. Benchmark module distribution and complete 90-question benchmark.

S8a. Benchmark module distribution

Benchmark module	Number of questions	Proportion (%)
mechanism	25	27.78
condition	17	18.89
concept	14	15.56
parameter	9	10.00
literature	9	10.00
comparison	7	7.78
structure_property	6	6.67
method	3	3.33
Total	90	100.00

S8b. Complete benchmark table format

See supplementary file: newest test_90.jsonl

This file provides the complete 90-question benchmark used in this study, including question identifiers, question texts, benchmark categories, gold-point references, and model responses where applicable.

Table S9. Per-question model scores and major-error labels.

See supplementary file: judge_detail_scores.json

This file provides the complete per-question scoring results for all evaluated models, including gold-point coverage, final scores, and major scientific error labels. And scores follow the gold-point scoring protocol: each covered gold point receives 1 point, one major scientific error deducts 1 point, and the final score is clipped at 0.

Table S10. Ablation analysis of RAG on the evidence-dependent benchmark subset.

Setting	Fine-tuned	RAG	n	Overall score	Major error
Base	No	No	18	2.278	2/18, 11.11%
Base + RAG	No	Yes	18	1.278	6/18, 33.33%
SFT only	Yes	No	18	1.333	2/18, 11.11%
SFT + RAG	Yes	Yes	18	1.500	4/18, 22.22%

Table S11. Representative case responses and gold-point evaluation.

See supplementary file: case_answer_summary.csv

This file provides the representative case responses and their gold-point-based evaluation, including covered gold points, missing gold points, major-error labels, and final evaluation results.

Table S12. Summary of expert manual review

Model	Mean expert score	Major error count	Major error rate
Llama-3.1-8B-Instruct base	1.818	1	9.1%
Qwen2.5-7B-Instruct	1.818	2	18.2%
NanoConfinementChat	2.364	0	0.0%
